# Refined ADME Profiles for ATC Drug Classes

**DOI:** 10.3390/pharmaceutics17030308

**Published:** 2025-02-28

**Authors:** Luca Menestrina, Raquel Parrondo-Pizarro, Ismael Gómez, Ricard Garcia-Serna, Scott Boyer, Jordi Mestres

**Affiliations:** 1 Chemotargets SL, Parc Cientific de Barcelona, Baldiri Reixac 4 (TR-03), 08028 Barcelona, Catalonia, Spain; 2 Institut de Quimica Computacional i Catalisi, Facultat de Ciencies, Universitat de Girona, Maria Aurelia Capmany 69, 17003 Girona, Catalonia, Spain

**Keywords:** physicochemical properties, ADME, pharmacokinetics, ATC classification, drug classes, generative chemistry, AI/ML models, AI drug discovery

## Abstract

**Background:** Modern generative chemistry initiatives aim to produce potent and selective novel synthetically feasible molecules with suitable pharmacokinetic properties. General ranges of physicochemical properties relevant for the absorption, distribution, metabolism, and excretion (ADME) of drugs have been used for decades. However, the therapeutic indication, dosing route, and pharmacodynamic response of the individual drug discovery program may ultimately define a distinct desired property profile. **Methods:** A methodological pipeline to build and validate machine learning (ML) models on physicochemical and ADME properties of small molecules is introduced. **Results:** The analysis of publicly available data on several ADME properties presented in this work reveals significant differences in the property value distributions across the various levels of the anatomical, therapeutic, and chemical (ATC) drug classification. For most properties, the predicted data distributions agree well with the corresponding distributions derived from experimental data across fourteen drug classes. **Conclusions:** The refined ADME profiles for ATC drug classes should be useful to guide the *de novo* generation of advanced lead structures directed toward specific therapeutic indications.

## 1. Introduction

Drug discovery and development is a lengthy, costly, and risky process, with current estimates of advancing a drug candidate to the market requiring on average 10 years, investments exceeding USD 1 billion, and failure rates of making it through all clinical phases above 90% [1,2,3,4]. Central to our ability to reduce time, expenditures, and attrition is to better understand and anticipate the pharmacokinetic properties of a drug, that is, its absorption, distribution, metabolism, and excretion (ADME) profile. Hansch et al. [5] were one of the first to note a relationship between the hydrophobic character of drugs, a property that can be estimated by calculating the octanol–water partition coefficient (logP), and their ability to passively cross the blood–brain barrier (BBB). Later, van de Waterbeemd and Kansy [6] established a correlation between the hydrogen bonding capacity of drugs, estimated as the polar surface area (PSA) occupied by nitrogen and oxygen atoms and the polar hydrogens attached to them, and their passive BBB penetration.

Those early works laid the foundation of the “rule of five” by Lipinski et al. [7,8] that established upper-bound criteria for the molecular size (molecular weight ≤ 500), hydrophobicity (clogP ≤ 5), and hydrogen bonding capacity (hydrogen bond donors ≤5 and hydrogen bond acceptors ≤ 10) of successful small-molecule drugs. Molecules with exceeding values of any two or more of those four property rules were deemed likely to possess poor solubility and permeability. For the past twenty-five years, these simple property-based rules have offered guidance to medicinal chemists on designing small molecules with likely better absorption and distribution [9,10].

With rising amounts of data available, the advent of artificial intelligence (AI)/machine learning (ML) methods is having a significant impact in drug discovery [11]. A most revealing recent example of this paradigm shift is the use of a generative AI-driven pipeline to produce a small-molecule inhibitor of a novel target for idiopathic pulmonary fibrosis within 18 months, with good pharmacokinetic, safety, and tolerability profiles in phase I clinical trials [12]. The success of such generative drug discovery programs [13] involves multi-property optimization of large generated molecular libraries. Within this framework, accurate ML models of ADME properties are an essential component that allows for substantially reducing the need for costly and time-consuming preclinical experiments [14,15,16]. The implementation of ADME models in industrial settings [17,18,19,20,21] offers a unique perspective of their performance and impact in a diversity of drug discovery projects.

This notwithstanding, general property rules for drugs may poorly reflect the subtle ADME differences required by indication-specific drug classes. For example, Mahar Doan et al. [22] and Adenot and Lahana [23] noticed significant differences with some molecular properties (hydrogen bond donor count, clogP, PSA, and number of rotatable bonds) between central nervous system (CNS) and non-CNS drugs. CNS drugs were generally found to be smaller, less polar, and less flexible than non-CNS drugs, which resulted in improved passive blood–brain barrier permeation. Leeson and Davis [24] analyzed the molecular properties of drugs across six therapeutic areas and showed that anti-infectives and CNS drugs had the most extreme property profiles. Altogether, the results from these early studies highlight the need to continuously survey and update the property profiles of drug classes.

This work introduces our efforts to build a computational pipeline for the construction and evaluation of AI/ML models for physicochemical and ADME properties of small molecules. The variability in the property value distributions across different drug classes/therapeutic indications is analyzed, and potential applications for guiding the generation of novel chemical structures are briefly discussed.

## 2. Materials and Methods

### 2.1. Collection and Classification of Marketed Drugs

The list of drugs considered in this work contains all small-molecule drugs with a known chemical structure that are present in the anatomical therapeutic chemical (ATC) classification system [25]. Table 1 collects a summary of the number of drugs assigned to the anatomical or pharmacological level of the ATC classification (ATC first level). The full list contains 2367 unique drugs. Note that some drugs may be assigned to multiple ATC codes, and thus the sum of the number of small-molecule drugs assigned to each ATC first level exceeds the total number of unique drugs.

### 2.2. Data Collection for Physicochemical and ADME Properties

Eight physicochemical properties known to be relevant for ADME property modeling were selected (Table 2). Out of them, six were calculated using RDKit version 2022.09.5 [26], namely, molecular weight (MW), PSA, number of hydrogen bond acceptors (HBA) and donors (HBD), number of rotatable bonds (ROTB), and logP (AlogP). For the two remaining properties, solubility (logS) and the acid–base dissociation strength (pKa), experimentally available data, for both small-molecule marketed medicines included in the ATC classification (drugs) and any other small molecules not being an approved marketed drug (non-drugs), were collected from the scholarly literature [27,28,29,30,31,32,33,34,35,36,37,38,39,40,41,42,43] and subsequently curated and integrated in a single data source. For molecules with multiple pKa values, only the strongest acidic or basic macroscopic pKa value was considered. Amphoteric molecules were excluded from the pKa set.

A selection of seven ADME properties is also considered in this work (Table 3). They cover absorption processes, human intestinal absorption (HIA), efflux ratio (logER), distribution processes, passive blood–brain barrier permeation (logBB), plasma protein binding (PPB), the volume of distribution in a steady state (logVDss), excretion processes, clearance rate (logCL), and half-life (logHL). Publicly available experimental data for these properties were assembled from e-Drug3D [44], DDPD 1.0 [45], and PhaKinPro [46] that complemented, for both drugs and non-drugs, data published in the scholarly literature [34,35,47,48,49,50,51,52,53,54,55,56,57,58,59,60,61,62,63,64,65,66,67].

### 2.3. Model Generation for Physicochemical and ADME Properties

Each molecular structure was mathematically represented by a list of 207 1D and 2D descriptors provided by RDKit [26] that served as input vectors to generate all property models. Prior to model generation, for each property, molecules were divided into two subsets: 85% for model training and validation, and 15% for final model evaluation. To best approach the real-case scenarios that the models will face upon deployment [68], the latter was further subdivided into two holdout sets containing, on one side, molecules selected from a scaffold-based split and, on the other side, the set of least-similar molecules. The scaffold-based split (7.5% of the holdout set) is a commonly used method for partitioning molecules with the same Murcko scaffold [69] into groups and then assigning these groups to different subsets (training and test sets) [70,71,72]. Compared to random splitting, scaffold-based splits tend to be a more representative split with regards to test generalization. In contrast, the least-similar split (the other 7.5% of the holdout set) is composed of those molecules having the largest pairwise Euclidean distances to their closest neighbor. Since it is well known that distance to the training set severely affects model performance [68], the least-similar split is designed to offer an even closer real-world testing scenario than the scaffold-based split.

Training and validation on the 85% of all property data were conducted using a 5-fold cross-validation (5FCV) process. The 5FCV process divides this portion of the dataset into 5 equal-sized random splits, iteratively trains the models on 80% of the data (68% of the total data), and uses the remaining 20% (17% of the total data) as the validation set. Seven types of ML algorithms were used to construct property models, namely, support vector regressor (SVR), random forest regressor (RFR), extra trees regressor (ETR), gradient boosting regressor (GBR), extreme gradient boosting regressor (XGBR), categorical boosting regressor (CBR), and adaptive boosting regressor (ABR). At each iteration, model performance was evaluated with the mean absolute error (MAE) and the root mean squared error (RMSE), using the standard deviation of each metric to measure the stability of the process and assess whether randomly splitting the data can be a source of bias [73]. The PyCaret library [74] was applied to compare performance metrics and select the most suitable models at this stage. Subsequently, for each property, the top five models were further optimized using hyperparameter optimization (HPO) methods available in PyCaret [74] and FLAML [75], with a time limit set to one hour.

**Figure 1:**
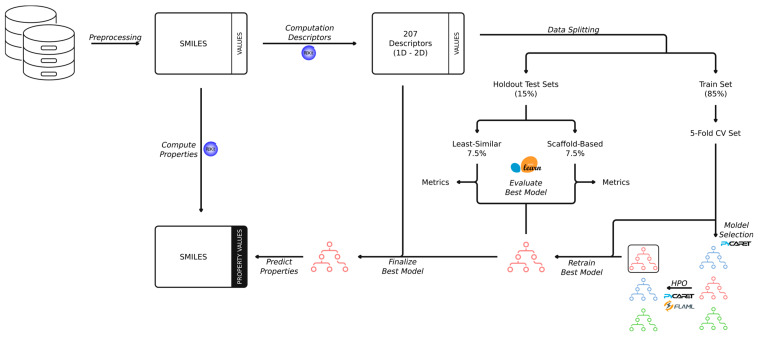
The PreCogs workflow for building machine learning models includes data collection and curation, descriptor computation from SMILES representations, data splitting, model selection and optimization via cross-validation, evaluation using holdout sets, and final model training on all available data.

The best ML algorithm leading to the best property model performance coming out of HPO is then retrained with the full set of training and validation data (85% of the total data) and applied to the two holdout sets for final model evaluation. An outline of the entire modeling pipeline is presented in Figure 1. All property models are integrated into our PreCogs package 2024.

## 3. Results and Discussion

### 3.1. Data Distributions of Drugs Versus Non-Drugs

**Figure 2:**
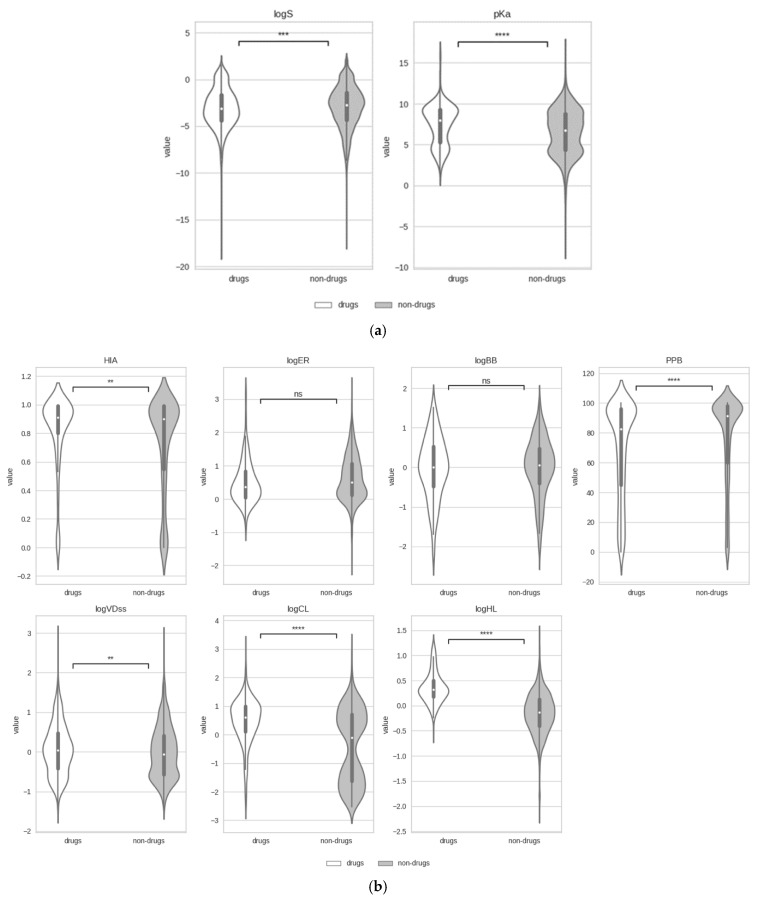
Violin plots of the distributions of (**a**) two physicochemical properties and (**b**) seven ADME properties for drugs and non-drugs. The white dot in each distribution marks the median value. Statistical significance is assessed with a two-sided Mann–Whitney–Wilcoxon test, with p-values annotated as follows: ns: 0.05 < *p* ≤ 1, **: 0.001< *p* ≤ 0.01, ***: 0.0001< *p* ≤ 0.001, ****: *p* ≤ 0.0001.

Distinguishing between the tiny portion of chemical space optimized for safe therapeutical action (drugs) and the remaining synthesizable molecules (non-drugs) has been a central theme in computer-aided drug design. In this respect, it was shown that drugs can be mostly distinguished from non-drugs in terms of 1D and 2D structural descriptors [76,77]. However, differences between the two sets in terms of property distributions may be more subtle. The experimental data distributions of physicochemical and ADME properties for the number of drug and non-drug molecules specified in Table 2 and Table 3, respectively, are presented in Figure 2.

Both solubility (logS) and the acid dissociation constant (pKa) are key properties affecting the ADME profile of drugs. Solubility has a direct impact on absorption and bioavailability, as poorly soluble compounds may struggle to reach therapeutic concentrations in the body. The acid dissociation constant reflects the ionization state of a molecule, which in turn influences its lipophilicity, solubility, protein binding, and ability to cross the plasma membrane. As can be observed in Figure 2a, differences in the distributions of both logS and pKa for drugs and non-drugs, although statistically significant, are visually very subtle. Interestingly, drugs appear to be slightly less soluble than non-drugs. This finding could be explained by considering that, compared to non-drugs, drugs have been optimized for potency. Also, in line with previous observations, drugs have on average higher pKa values; that is, they are found to be slightly more basic than non-drugs, a trend that could be emphasized by the fact that almost 20% of the drugs considered in this work are CNS drugs [78].

Regarding ADME properties (Figure 2b), the largest statistical differences between drugs and non-drugs are found in the distributions for plasma protein binding (PPB), clearance (logCL), and half-life (logHL). Most drugs have high PPB values (80–100%) [79], but interestingly, non-drugs seem to have on average an even higher affinity for plasma proteins. The lower PPB values for drugs relative to non-drugs is what makes drugs more available for therapeutic action. Additionally, drugs tend to have clearly higher logCL and logHL values compared to non-drugs. These two excretion properties allow drugs to remain active in the body long enough to exert their therapeutic effect while still being eliminated at a rate that minimizes the risk of accumulation and potential toxicity. In contrast, the distributions of efflux ratio (logER) and passive blood–brain barrier penetration (logBB) available for drugs were found to be comparable to those for non-drugs. This result may not come as a surprise if one considers that these absorption and distribution properties may only be relevant to certain drug classes, and thus drugs in general may not have been necessarily optimized for them.

The comparative analysis of physicochemical and ADME property distributions between drugs and non-drugs supports the fact that drugs possess subtle yet distinct characteristics that differentiate them from other chemicals or small molecules. However, the distributions of some ADME properties (such as logBB) of key importance for certain drug classes (such as CNS drugs) remain undistinguishable when comparing all drugs against non-drugs. Deconvoluting the general analysis of all drugs into drug classes may reveal distinct property distributions better aligned with therapeutic indications.

### 3.2. Data Completeness Across Drug Classes

An analysis of the current coverage of experimental property data across drug classes reveals a generally low and uneven completeness in both directions of the class–property matrix (Table 4). Coverage of the different property data across each drug class ranges between 19% (L, antineoplastic and immunomodulating agents) and 34% (N, central nervous system drugs), with no drug class consistently covered more or less than others. In contrast, coverage of the various drug classes across properties is more variable. While solubility and plasma protein binding contain data that cover more than 50% of the drugs considered in this study, less than 10% of the drugs have available experimental data for passive blood–brain barrier penetration, efflux ratio, and half-life.

For some properties, the low coverage of the data collected responds to a decision to use only quality datasets. For example, half-life data were limited to the set published by Fan et al. [67], the source of choice also by other platforms such as ADMETlab 3.0 [80]. This notwithstanding, relatively higher coverages of data are achieved in drug classes for which the property is particularly important. In the case of half-life data, even though the average coverage is 8.5%, the coverage for anti-infectives for systemic use (drug class J) is 23.5%. Along the same lines, while 18% of CNS drugs have data for passive blood–brain barrier penetration, the average drug coverage is only 6.0%. Overall, the low levels of experimental data completeness observed across drug classes highlight the need for constructing property models that provide full coverage through computed properties.

### 3.3. Physicochemical Properties for Drug Classes

The performance metrics of the best models derived for solubility and pKa are provided in Table 5. For solubility, the ML algorithm that returned the best model is an extreme gradient boosting regressor, resulting in MAE and RMSE values of 0.55 ± 0.01 and 0.83 ± 0.03, respectively, after five-fold cross-validation. Final model evaluation against scaffold-based and least-similar splits returned MAE values of 0.73 and 1.42, respectively. For the scaffold-based split, albeit with different datasets, the MAE value from PreCogs (0.73) is comparable to the values reported recently from Chemprop-RDKit and MolE (0.76 ± 0.03 and 0.78 ± 0.02, respectively) [81]. Using a scaffold-based split with the same dataset, MAE values between 0.91 and 1.12 were obtained with a variety of publicly available web services, including ADMETlab 3.0 [80], admetSAR 3.0 [82], Deep-PK [83], ADMET-AI [84], and SwissADME [85]. For the acid dissociation constant, the extra trees regressor outperformed the other ML algorithms. The MAE and RMSE values obtained for the best model are 1.03 ± 0.03 and 1.68 ± 0.08, respectively. Slightly higher MAE values were attained for the scaffold-based (1.16) and least-similar (1.36) splits. In comparison, for the scaffold-based split, admetSAR 3.0 [82] and Deep-PK [83] returned MAE values of 1.07 and 2.44, respectively. Examples of the best (lowest MAE values) and worst (highest MAE values) logS and pKa predictions are provided in Supplementary Tables S1 and S2.

**Figure 3:**
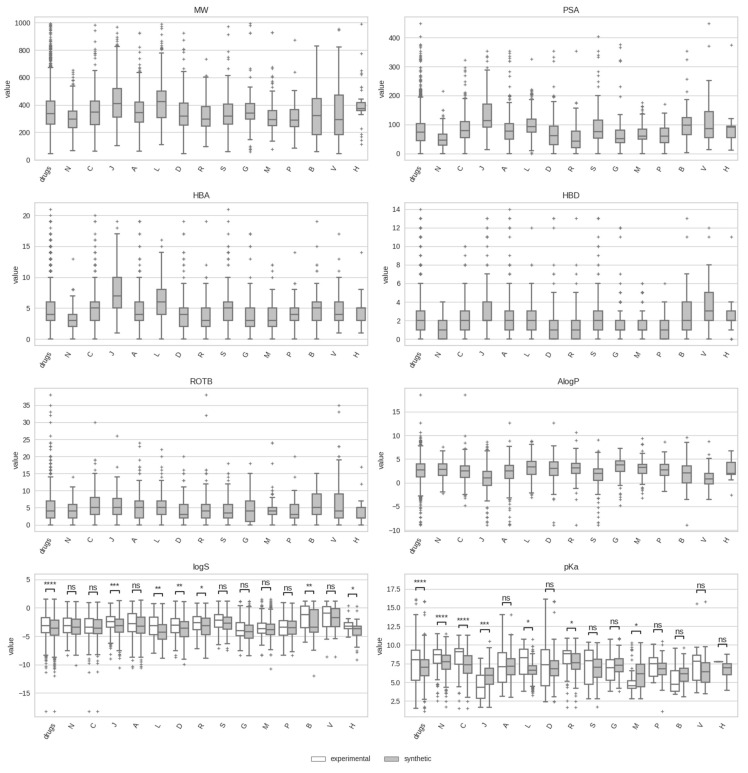
Boxplots illustrating the distributions of physicochemical properties for various drug classes (ATC 1st level). Plots for MW, PSA, HBA, HBD, ROTB, and AlogP show exclusively the distributions obtained with calculated values, whereas plots for logS and pKa show the comparative distributions between experimental and computational data. Each boxplot displays the median, upper and lower quartiles, whiskers extending to 1.5 times the interquartile range, and individual points marking outliers. Statistical significance was assessed with a two-sided Mann–Whitney–Wilcoxon test, with p-values annotated as follows: ns: 0.05 < *p* ≤ 1, *: 0.01< *p* ≤ 0.05, **: 0.001< *p* ≤ 0.01, ***: 0.0001< *p* ≤ 0.001, ****: *p* ≤ 0.0001.

Figure 3 displays the data distributions for the eight physicochemical properties across the fourteen drug classes considered (ATC first level). As can be observed, there are variations when property distributions for all drugs are compared to the respective distributions for various drug classes. For example, antineoplastic and immunomodulating agents (drug class L) exhibit the highest values in the boxplots of molecular weight (MW) and polar surface area (PSA), consistent with the large and complex nature of many oncology drugs. High PSA values within the boxplot are typically associated with low passive blood–brain barrier permeability, a property that this class of drugs, in most cases, does not need to possess. Conversely, CNS drugs (drug class N) tend to have low MW and PSA value distributions to favor their ability to cross the blood–brain barrier, an essential property for CNS drugs. Anti-infectives for systemic use (drug class J), which includes penicillins and cephalosporins, have some of the highest values within the boxplots for MW, PSA, HBA, and HBD, resulting in one of the distributions with the lowest AlogP values being in the corresponding boxplot. Previous studies suggest that a low-to-moderate lipophilicity range (logP ~ 0–2.5) is optimal for passive cytoplasmic permeation of antibacterial agents, enabling them to penetrate bacterial outer membranes effectively without becoming trapped in lipid-rich environments. This property is especially relevant for antibiotics targeting Gram-negative bacteria, where outer membrane permeability can significantly impact efficacy [86,87].

With regards to solubility and the acid dissociation constant, for most drug classes (eight out of fourteen), distributions obtained from the limited experimental data available for both properties are statistically no different than distributions derived from calculated data obtained from the property models, the latter having the advantage of offering full coverage of all drugs within each class. For solubility, drugs belonging to the ATC first-level class of blood and blood-forming organs (drug class B) exhibit the highest logS values within the boxplot. High solubility can be considered advantageous for drugs that act in the bloodstream, as it ensures effective dissolution in blood plasma (primarily an aqueous medium) and supports systemic circulation. In contrast, drugs of the genitourinary system and sex hormones (drug class G) are among the classes with the lowest logS values within the boxplot, indicative of highly lipophilic compounds. For the acid dissociation constant, anti-infectives for systemic use (drug class J) exhibit the lowest pKa values, a trend likely linked to the acidic nature of many antibiotics [88].

### 3.4. ADME Properties for Drug Classes

The performance metrics of the best models derived for all seven ADME properties considered in this work are provided in Table 6. In all cases, the ML algorithm leading to the best model is an extra trees regressor. For the five ADME properties in logarithmic units (logER, logBB, logVDss, logCL, and logHL), MAE values range from 0.16 ± 0.01 (logHL) to 0.56 ± 0.01 (logCL), whereas for the two ADME properties in percentage units, MAE values range from 9.8 ± 0.4 (HIA) to 10.7 ± 0.6 (PPB). The corresponding MAE values in the final model evaluation range from 0.20 (logHL) to 0.69 (logCL) and from 14.6 (HIA) to 15.4 (PPB) for the scaffold-based split, and from 0.20 (logHL) to 0.85 (logCL) and from 24.1 (HIA) to 16.1 (PPB) for the least-similar split. Examples of the best (lowest MAE values) and worst (highest MAE values) predictions for HIA, logER, logBB, PPB, logVDss, logCL, and logHL are provided in Supplementary Tables S3–S9.

Using scaffold-based splitting, MAE values of 0.11 and 0.23 for logVDss were obtained with ADMETlab 3.0 [80] and Deep-PK [83], respectively, compared to the MAE value from PreCogs of 0.30. For logCL, with an MAE value from PreCogs of 0.69, the same web services returned MAE values of 1.02 and 1.03. Spearman correlation coefficients for logVDss and logHL reported from MolE are 0.64 ± 0.01 and 0.58 ± 0.03, compared to the values of 0.65 and 0.80 obtained with PreCogs. For PPB, the best reported MAE values come from MolE [81] (7.2 ± 0.2), MapLight + GNN [81] (7.5 ± 0.1), and ADMETlab 3.0 [80] (8.2). MAE values between 13.6 and 19.2, in the range of PreCogs (15.4), are obtained with admetSAR 3.0 [82], Deep-PK [83], and ADMET-AI [84].

**Figure 4:**
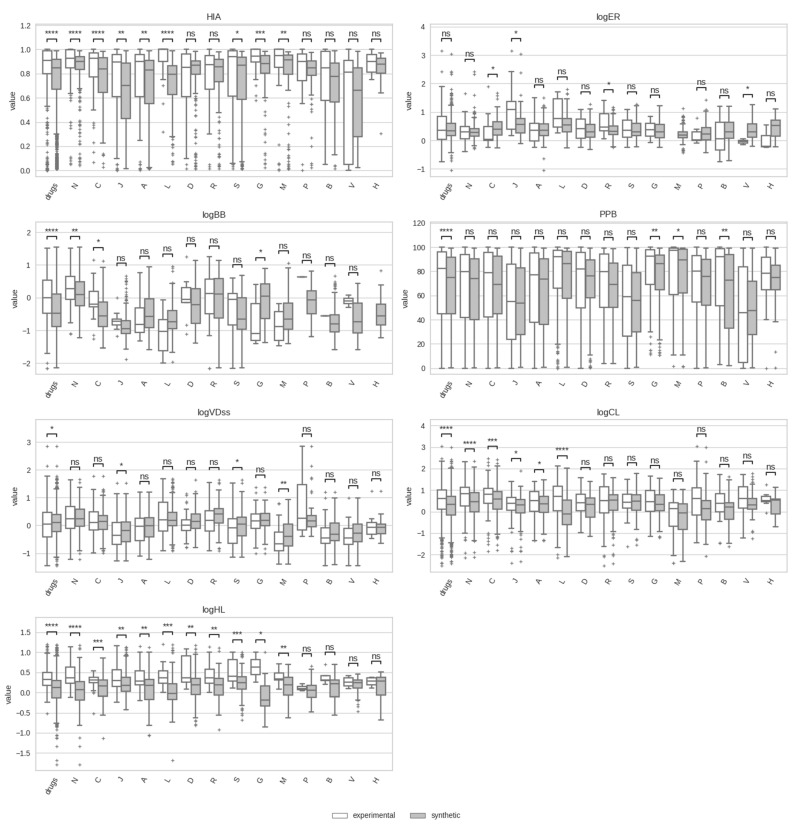
Boxplots illustrating the distributions of ADME properties for various drug classes (ATC 1st level). Each boxplot displays the median, upper and lower quartiles, whiskers extending to 1.5 times the interquartile range, and individual points marking outliers. Statistical significance was assessed with a two-sided Mann–Whitney–Wilcoxon test, with p-values annotated as follows: ns: 0.05 < *p* ≤ 1, *: 0.01< *p* ≤ 0.05, **: 0.001< *p* ≤ 0.01, ***: 0.0001< *p* ≤ 0.001, ****: *p* ≤ 0.0001.

Figure 4 displays the experimental and calculated data distributions for the seven ADME properties across the fourteen drug classes considered (ATC first level). Within each ADME property, significant variations among drug classes are observed. The three distribution properties (logBB, PPB, and logVDss) appear to be particularly sensible to drug classes compared to absorption and excretion properties. Interestingly, the best correspondence between experimental and computational data distributions is also obtained for these three distribution properties, with ten (logBB), eleven (PPB), and eleven (logVDss) drug classes out of all fourteen classes showing no statistical difference between the two data distributions. In contrast, only in four and six drug classes are the experimental data distributions not significantly different from the calculated data distributions for logHL and HIA, respectively.

A closer examination of the data distributions for HIA reveals that drug classes J, B, and V have the lowest values within their respective boxplots, likely reflecting a variety of therapeutic needs and routes of administration. For example, within drug class J, some antibiotics are specifically formulated to have poor absorption. This allows them to act locally within the gastrointestinal (GI) tract [89,90], whereas others are administered parenterally to bypass the GI tract entirely [91].

Drug classes L and J exhibit high values within the boxplot for efflux ratio (logER), possibly linked to efflux-mediated resistance. For antineoplastic agents (drug class L), high logER values within the boxplot are associated with resistance mechanisms where drugs are actively expelled from cancer cells by efflux proteins like P-glycoprotein, a key factor in multidrug resistance [92,93]. Similarly, in bacterial cells, efflux pumps play a crucial role in antibiotic resistance, particularly among Gram-negative bacteria [94,95].

The boxplots with the highest logBB values contained in them are observed in drug classes N, R, and G, reflecting the general need of these drugs for membrane penetration to reach the site of action of their primary targets, mainly G protein-coupled receptors for classes N and R and nuclear receptors for class G. This high permeability is especially important for many CNS drugs (N), as they need to cross the blood–brain barrier or the blood–cerebrospinal fluid barrier to reach their intended CNS targets. Similarly, drugs of the respiratory system (R) must pass the alveolar barrier for effective lung absorption, and drugs of the genitourinary system and sex hormones (G) often require nuclear penetration for hormone–receptor interactions within target cells.

Drug classes M and B have logVDss distributions with values within the boxplots slightly lower than those of the other drug classes, likely connected to their relatively higher PPB values. The inverse relationship between logVDss and PPB is well established. Drugs that bind extensively to plasma proteins generally have a smaller volume of distribution because a relatively large proportion of the dose remains confined within the plasma [96,97,98]. This property is especially relevant for drugs acting primarily within the circulatory system, as high plasma protein binding limits the rate of diffusion into tissues.

Low clearance (logCL) values are observed in the boxplots of drug classes L and M. For antineoplastic agents (drug class L), low baseline clearance has been associated with improved clinical outcomes, as reduced clearance rates allow for prolonged drug exposure, potentially enhancing therapeutic efficacy in cancer treatments [99]. The class of musculoskeletal drugs (M), often administered locally, may also show low clearance profiles, as localized delivery reduces the need for systemic circulation and thus impacts overall clearance rates [100,101].

Despite the clear variations observed in experimental ADME property distributions across drug classes, one may argue that the first level of the ATC classification offers sufficient granularity to capture the particular ADME requirements of certain drug subclasses. Naturally, ATC first-level classes include drugs with a wide diversity of mechanisms of action, many likely targeting a variety of tissues, and thus requiring essentially different ADME profiles. For example, drugs in class N can target proteins expressed in the brain or in the peripheral tissues. In the latter case, brain penetration would not be necessary. However, the population bias in class N of drugs targeting brain proteins may give the impression that high logBB values are generally required for all class N drugs.

**Figure 5:**
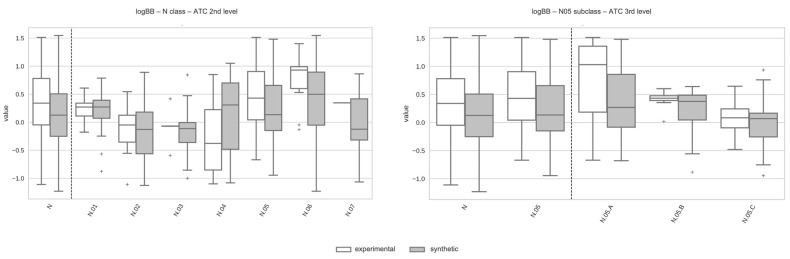
Boxplots illustrating the distributions of logBB for ATC 2nd-level (**left**) and ATC 3rd-level (**right**) subclasses from drug class N. Each boxplot displays the median, upper and lower quartiles, whiskers extending to 1.5 times the interquartile range, and individual points marking outliers.

To illustrate those aspects, Figure 5 shows the variability in both experimental and predicted logBB value distributions as we explore deeper levels of the ATC classification within the class of nervous system drugs (N). Going just one level down (ATC second level), we observe that, compared to the boxplot considering all class N drugs, logBB distributions for anesthetics (N01), analgesics (N02), and anti-Parkinson drugs (N04) have lower values than the corresponding distributions for psycholeptics (N05) and especially psychoanaleptics (N06), populated mainly with drugs targeting proteins expressed in the brain. As we move further down (ATC third level) from subclass N05 drugs (psycholeptics), we see that, taking the boxplot from all subclass N05 drugs as reference, logBB distributions for hypnotics and sedatives (N05C) have lower values than the corresponding distribution for antipsychotics (N05A), composed of drugs targeting G protein-coupled receptors expressed in the brain. These results highlight the need to go as deep as the availability of experimental data allows into the ATC classification to obtain refined ADME profiles for drug classes.

## 4. Conclusions

This work establishes a reference framework for modeling 15 physicochemical and ADME properties included in our PreCogs package. The current main limitation is drug space coverage of available experimental property data. More experimental data covering a wider portion of drugs within classes will likely improve the performance and generalizability of the present models.

From a methodological viewpoint, an interesting observation is the fact that the ML algorithm leading to the best model in most cases was an extra trees regressor (ETR). The best model for solubility (logS) was the only one using an extreme gradient boost regression (XGBR). ETR is an ensemble ML approach similar to random forest (RF), in which multiple decision trees are trained and their predictions are ultimately aggregated. ETR differs from RF in two key aspects: it uses the entire dataset to train each individual decision tree and randomly selects feature split values, making it computationally efficient [102]. XGBR is a robust implementation of the gradient boosting algorithm where each tree is trained sequentially to correct the residuals of the previous trained ones, optimized for high efficiency, speed, and scalability. Although ETR is less demanding in terms of computational cost, XGBR’s architecture is designed to handle large-scale data better than ETR [103]. This difference could explain why our modeling pipeline identified ETR as the best performing model for nearly all properties except for logS, which has the largest dataset, nearly twice as many molecules as the second largest (pKa). The size of the logS dataset likely benefits from XGBR’s gradient boosting framework, making it the top performer for logS despite ETR’s overall efficiency in handling smaller datasets.

The present analysis of experimental and computational data for 15 physicochemical and ADME properties across 14 different drug classes reveals several trends that highlight the specificities of each class. For example, an inverse relationship between hydrophobicity (AlogP) and solubility (logS) values is observed across drug classes. As AlogP increases, logS tends to decrease. Two other inverse relationships observed are between molecular weight (MW) and polar surface area (PSA) and passive blood–brain barrier permeability (logBB). Both MW and PSA are key determinants of the ability of a molecule to cross biological barriers, such as logBB. Larger MW and higher PSA values tend to hinder the ability of a drug to passively cross the blood–brain barrier (low logBB). Finally, another inverse relationship between the volume of distribution in a steady state (logVDss) and plasma protein binding (PPB) is observed, reflecting the fact that drugs that bind extensively to plasma proteins typically exhibit a smaller volume of distribution as they remain largely confined within the plasma. Improving our ability to predict properties for drug classes can have an impact on multiple aspects of drug design, from narrowing the properties for certain routes of administration to directing the property profiles toward the intended pharmacological effects and therapeutic indications.

The observed differences between experimental (measured) and calculated (predicted) physicochemical and ADME properties for drug classes reflect not only the subtleties in the modeling approach but also the distinct nature of the datasets being compared. For properties like solubility and plasma protein binding, the distributions obtained with computed data are comparable to those derived from experimental data, likely due to the broader data coverage for these properties, allowing the models to make more accurate predictions, even when applied to a diverse set of drugs. Similarly, logHL has one of the lowest data coverages, which results in calculated distributions being markedly different than those obtained from the limited experimental data available. These results underscore the importance of having access to larger, wider, high-quality experimental datasets to ensure that predictions derived from computational models align with the expected properties within drug classes.

Physicochemical and ADME property distributions are the result of decades of successful drug development programs against a backdrop of hundreds of unsuccessful attempts. As such, they represent the properties most likely to result in useful drugs. However, the constraints of the therapeutic indication, dosing route, target tissue, and desired pharmacodynamic response will ultimately determine the final property profile in an individual program. Indeed, significant variations in several properties across different drug classes have been observed in this work and reproduced with ML models, resulting in more refined definitions of drug property boundaries within therapeutic indications.

Besides gaining a better understanding of the ADME profile of drug classes, the construction of a methodological pipeline for ML model building and the evaluation of physicochemical and ADME properties for small molecules is a key component in today’s computational drug discovery platforms aiming at generating advanced lead compounds. The integration of these models into a generative chemistry engine should act as a useful compass to efficiently guide the design of novel synthetically feasible small molecules with ADME profiles that best fit known property distributions within specific therapeutic classes.

## Figures and Tables

**Table 1:** The letter identifier, drug class definition, and number of small-molecule drugs of the fourteen main anatomical or pharmacological groups of the ATC classification (ATC 1st level).

ATC 1st Level	Definition	Number of Small-Molecule Drugs
N	Nervous System	452
C	Cardiovascular System	323
J	Anti-Infectives for Systemic Use	298
A	Alimentary Tract and Metabolism	284
L	Antineoplastic and Immunomodulating Agents	268
D	Dermatologicals	217
R	Respiratory System	191
S	Sensory Organs	164
G	Genitourinary System and Sex Hormones	144
M	Musculo-Skeletal System	130
P	Antiparasitic Products, Insecticides and Repellents	89
B	Blood and Blood Forming Organs	87
V	Various	77
H	Systemic Hormonal Preparations, Excl. Sex Hormones and Insulins	37

**Table 2:** The acronym, definition, total counts of drugs and non-drugs, and data sources for the selection of eight physicochemical properties considered in this work.

Property	Definition	Number of Drugs	Number of Non-Drugs	Sources
MW	Total mass of a compound’s atoms, expressed in Daltons (g/mol)	-	-	Computed with RDKit v2022.09.5 [26]
PSA	Total surface area of polar atoms, typically oxygen and nitrogen, including attached hydrogens	-	-	Computed with RDKit v2022.09.5 [26]
HBA	Number of atoms capable of accepting hydrogen bonds	-	-	Computed with RDKit v2022.09.5 [26]
HBD	Number of atoms capable of donating hydrogen bonds	-	-	Computed with RDKit v2022.09.5 [26]
ROTB	Number of single, non-ring bonds which allow for free rotation around themselves	-	-	Computed with RDKit v2022.09.5 [26]
AlogP	Logarithmic (base 10) transformation of the partition coefficient between octanol and water	-	-	Computed with RDKit v2022.09.5 [26]
logS	Logarithmic (base 10) transformation of solubility (mol/L)	1256	17,001	[27,28,29,30,31,32,33,34,35,36,37,38,39]
pKa	Logarithmic measure of the acid–base dissociation strength	406	9234	[40,41,42,43]

**Table 3:** The acronym, definition, total counts of drugs and non-drugs, and data sources for the selection of seven ADME properties considered in this work.

Property	Definition	Number of Drugs	Number of Non-Drugs	Sources
Absorption				
HIA	Percentage of a compound absorbed in the human intestine	512	590	[47,48]
logER	Logarithmic (base 10) of the efflux ratio determined in Caco-2 cell permeability assays	93	2644	[22,35,49,50]
Distribution				
logBB	Logarithmic (base 10) transformation of the brain-to-blood concentration ratio	141	439	[51,52,53,54,55]
PPB	Percentage of a compound bound to plasma proteins in the bloodstream	1259	3565	[34,44,45,46,56,57,58,59]
logVDss	Logarithmic (base 10) transformation of the theoretical volume needed to contain the total amount of a compound at plasma concentration in the steady state (L/kg)	784	1604	[59,60,61,62]
Excretion				
logCL	Logarithmic (base 10) transformation of the total clearance rate (mL/min/kg)	755	1512	[59,61,63,64,65,66]
logHL	Logarithmic (base 10) transformation of half-life (h)	202	3266	[67]

**Table 4:** Percentage of available experimental physicochemical and ADME property data across drug classes (ATC class).

ATC Class	Physicochemical Properties		ADME Properties							Global
	logS	pKa	HIA	logBB	logCL	logER	logHL	logVDss	PPB	
A	57.75	15.49	19.72	2.46	28.52	3.52	6.69	28.17	43.31	22.85
B	49.43	19.54	13.79	1.15	21.84	3.45	5.75	28.74	48.28	21.33
C	65.02	22.29	34.06	5.88	39.94	6.50	9.29	42.41	60.37	31.75
D	63.13	20.28	16.13	5.07	16.59	0.92	5.07	18.43	39.17	20.53
G	52.78	15.28	19.44	3.47	29.17	1.39	1.39	31.25	46.53	22.30
H	48.65	2.70	21.62	0.00	27.03	8.11	10.81	29.73	48.65	21.92
J	48.32	10.40	18.79	3.02	46.31	3.69	23.49	48.99	61.07	29.34
L	23.51	5.60	8.96	2.61	30.22	2.99	4.10	29.85	65.30	19.24
M	66.92	31.54	34.62	3.08	35.38	0.00	10.77	35.38	58.46	30.68
N	65.04	27.88	33.63	17.92	41.37	5.53	7.96	43.36	62.83	33.95
P	70.79	6.74	16.85	1.12	16.85	6.74	4.49	16.85	48.31	20.97
R	60.21	23.04	23.04	8.38	25.13	4.71	5.76	24.61	43.46	24.26
S	67.68	22.56	26.83	7.93	30.49	4.27	13.41	35.37	55.49	29.34
V	58.44	16.88	18.18	5.19	28.57	2.60	9.09	29.87	40.26	23.23
Global	53.06	17.15	21.63	5.96	31.90	3.93	8.53	33.12	53.19	

**Table 5:** The performance of the best property models expressed as the mean ± standard deviation of the MAE and RMSE for the best ML algorithm after 5-fold cross-validation was applied to 85% of each dataset and the MAE and RMSE for the updated best ML model against two holdout sets accounting for 15% of each dataset. The ML algorithms leading to the best models for logS and pKa are extreme gradient boosting regressor and extra trees regressor, respectively.

	Training and Validation (85%)		Evaluation (15%)			
Property	5-Fold Cross-Validation		Scaffold-Based Set		Least-Similar Set	
	MAE	RMSE	MAE	RMSE	MAE	RMSE
logS	0.55 ± 0.01	0.83 ± 0.03	0.73	1.07	1.42	1.94
pKa	1.03 ± 0.03	1.68 ± 0.08	1.16	1.71	1.36	1.80

**Table 6:** The performance of the best ADME models expressed as the mean ± standard deviation of the MAE and RMSE for the best ML algorithm after 5-fold cross-validation was applied to 85% of each dataset and the MAE and RMSE for the updated best ML model against two holdout sets accounting for 15% of each dataset. For all ADME properties, the ML algorithm leading to the best model is extra trees regressor.

	Training and Validation (85%)		Evaluation (15%)			
Property	5-Fold Cross-Validation		Scaffold-Based		Least-Similar	
	MAE	RMSE	MAE	RMSE	MAE	RMSE
HIA	9.8 ± 0.4	17.7± 1.2	14.6	20.1	24.1	30.0
logER	0.25 ± 0.01	0.40 ± 0.02	0.37	0.54	0.48	0.61
logBB	0.19 ± 0.03	0.32 ± 0.04	0.37	0.46	0.44	0.56
PPB	10.7 ± 0.6	16.9 ± 0.9	15.4	20.7	16.2	22.5
logVDss	0.10 ± 0.01	0.23 ± 0.02	0.30	0.42	0.42	0.55
logCL	0.56 ± 0.01	0.82 ± 0.02	0.69	0.88	0.85	1.02
logHL	0.16 ± 0.01	0.20 ± 0.01	0.20	0.26	0.20	0.26

## Data Availability

References to all sources of publicly available experimental data are provided.

## Supplementary Materials

The following supporting information can be downloaded at https://www.mdpi.com/article/10.3390/pharmaceutics17030308/s1, Table S1: Property: logS | ML algorithm: XGBR; Table S2: Property: pKa | ML algorithm: XTR; Table S3: Property: HIA | ML algorithm: XTR; Table S4: Property: logER | ML algorithm: XTR; Table S5: Property: logBB | ML algorithm: XTR; Table S6: Property: PPB | ML algorithm: XTR; Table S7: Property: logVDss | ML algorithm: XTR; Table S8: Property: logCL | ML algorithm: XTR; Table S9: Property: logHL | ML algorithm: XTR.
