# Supplementary material for: Refined ADME Profiles for ATC Drug Classes
Source: Pharmaceutics. 2025 Feb 28;17(3):308. doi: 10.3390/pharmaceutics17030308 (PMC11944659; doi:10.3390/pharmaceutics17030308)
Supplement: Supplementary file 1 [file pharmaceutics-17-00308-s001.zip › pharmaceutics-3350713-supplementary.pdf]

Supplementary Material

Refined ADME Profiles for ATC Drug Classes

Luca Menestrina [1], Raquel Parrondo-Pizarro [1,2], Ismael Gómez [1], Ricard Garcia-Serna [1], Scott Boyer [1] and Jordi Mestres [1,2,*]

[1] Chemotargets SL, Parc Cientific de Barcelona, Baldiri Reixac 4 (TR-03), 08028 Barcelona, Catalonia, Spain;
[2] Institut de Quimica Computacional i Catalisi, Facultat de Ciencies, Universitat de Girona, Maria Aurelia Capmany 69, 17003 Girona, Catalonia, Spain.
* Correspondence to: jordi.mestres@chemotargets.com (J.M.).

Table S1. Property: logS | ML algorithm: XGBR

Top3 predictions (lowest AE values)

**Training set**	**Scaffold-based set**		**Training set**	**Least-similar set**
Mol in training	Mol in test		Mol in training	Mol in test
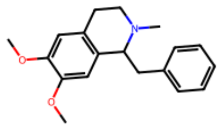<br>Exp logS: -3.649964<br>Similarity: 0.66	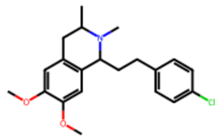<br>Exp logS: -5.416361<br>Pred logS: -5.415882		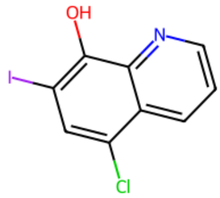<br>Exp logS: -1.822256<br>Similarity: 0.46	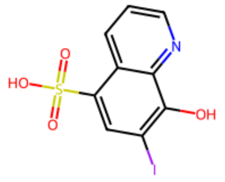<br>Exp logS: -2.200000<br>Pred logS: -2.200099
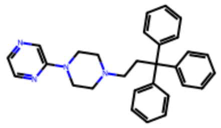<br>Exp logS: -6.517503<br>Similarity: 0.56	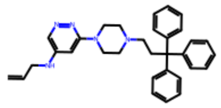<br>Exp logS: -7.384699<br>Pred logS: -7.384114		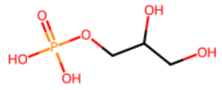<br>Exp logS: 0.764300<br>Similarity: 0.51	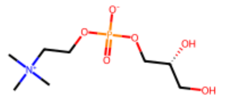<br>Exp logS: 0.589690<br>Pred logS: 0.588153
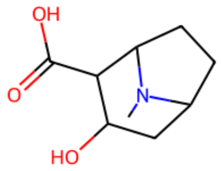<br>Exp logS: -0.020000<br>Similarity: 0.63	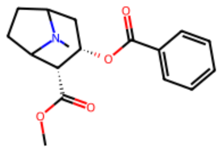<br>Exp<br>logS: -2.226700<br>Pred logS: -2.227477		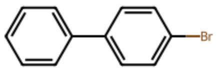<br>Exp logS: -5.552600<br>Similarity: 0.47	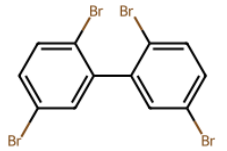<br>Exp logS: -8.060000<br>Pred logS: -8.057582

Bottom3 predictions (highest AE values)

**Training set**	**Scaffold-based set**		**Training set**	**Least-similar set**
Mol in training	Mol in test		Mol in training	Mol in test
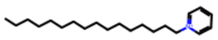<br>Exp logS: -0.486150<br>Similarity: 0.51	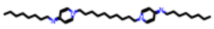<br>Exp logS: -1.642786<br>Pred logS: -7.277739		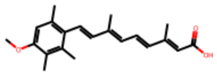<br>Exp logS: -6.651071<br>Similarity: 0.52	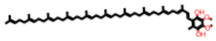<br>Exp logS: -18.217693<br>Pred logS: -7.325727
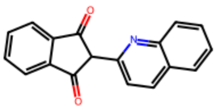<br>Exp logS: -4.701186<br>Similarity: 0.42	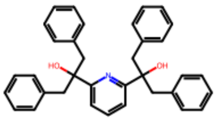<br>Exp logS: -9.630483<br>Pred logS: -4.555500		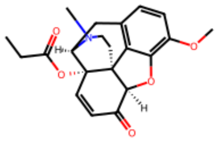<br>Exp logS: -2.791543<br>Similarity: 0.68	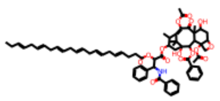<br>Exp logS: -17.415630<br>Pred logS: -7.354840
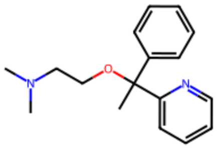<br>Exp logS: -2.820000<br>Similarity: 0.58	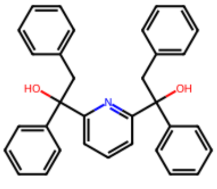<br>Exp logS: -8.587214<br>Pred logS: -3.816390		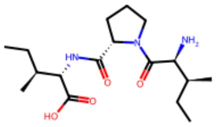<br>Exp logS: -2.560664<br>Similarity: 0.51	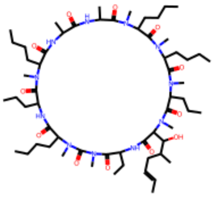<br>Exp logS: -11.997610<br>Pred logS: -1.983598

Table S2. Property: pKa | ML algorithm: XTR

Top3 predictions (lowest AE values)

**Training set**	**Scaffold-based set**		**Training set**	**Least-similar set**
Mol in training	Mol in test		Mol in training	Mol in test
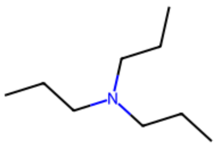<br>Exp pKa: 10.47<br>Similarity: 0.66	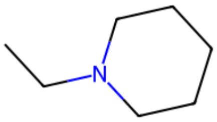<br>Exp pKa: 10.68<br>Pred pKa: 10.681105		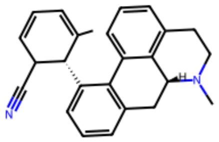<br>Exp pKa: 7.30<br>Similarity: 0.42	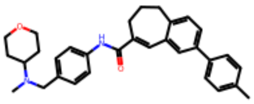<br>Exp pKa: 8.30<br>Pred pKa: 8.297558
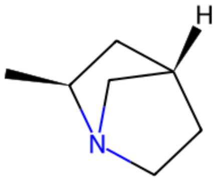<br>Exp pKa: 10.62<br>Similarity: 0.62	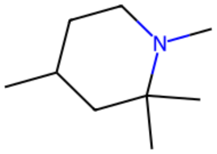<br>Exp pKa: 10.24<br>Pred pKa: 10.245079		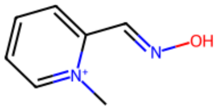<br>Exp pKa: 7.75<br>Similarity: 0.52	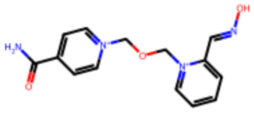<br>Exp pKa: 7.13<br>Pred pKa: 7.124582
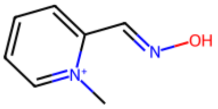<br>Exp pKa: 7.75<br>Similarity: 0.52	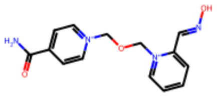<br>Exp pKa: 7.13<br>Pred pKa: 7.124582		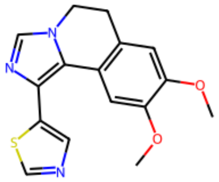<br>Exp pKa: 4.30<br>Similarity: 0.39	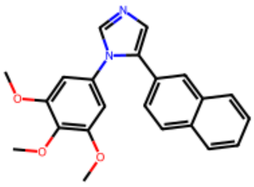<br>Exp pKa: 5.58<br>Pred pKa: 5.570520

Bottom3 predictions (highest AE values)

**Training set**	**Scaffold-based set**		**Training set**	**Least-similar set**
Mol in training	Mol in test		Mol in training	Mol in test
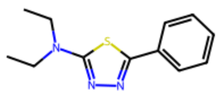<br>Exp pKa: 3.09<br>Similarity: 0.65	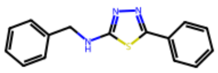<br>Exp pKa: 11.50<br>Pred pKa: 4.987414		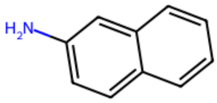<br>Exp pKa: 4.16<br>Similarity: 0.32	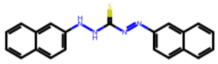<br>Exp pKa: 12.68<br>Pred pKa: 5.452964
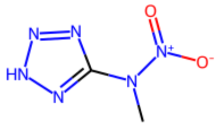<br>Exp pKa: 2.88<br>Similarity: 0.29	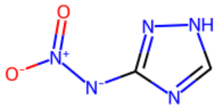<br>Exp pKa: 10.80<br>Pred pKa: 4.295941		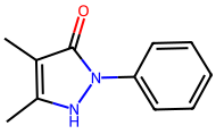<br>Exp pKa: 11.30<br>Similarity: 0.57	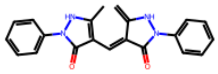<br>Exp pKa: 13.80<br>Pred pKa: 6.938822
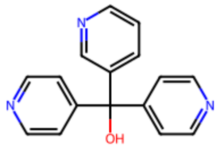<br>Exp pKa: 11.90<br>Similarity: 0.63	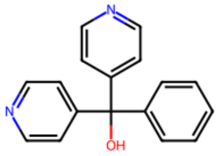<br>Exp pKa: 13.07<br>Pred pKa: 6.888510		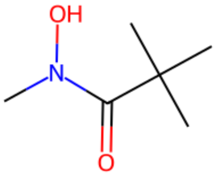<br>Exp pKa: 9.94<br>Similarity: 0.26	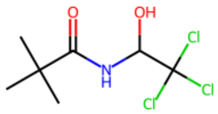<br>Exp pKa: 14.65<br>Pred pKa: 7.867859

Table S3. Property: HIA | ML algorithm: XTR

Top3 predictions (lowest AE values)

**Training set**	**Scaffold-based set**		**Training set**	**Least-similar set**
Mol in training	Mol in test		Mol in training	Mol in test
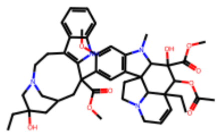<br>Exp HIA: 0.05<br>Similarity: 0.54	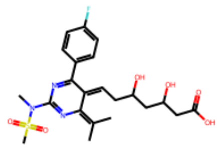<br>Exp HIA: 0.50<br>Pred HIA: 0.501577		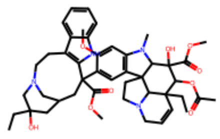<br>Exp HIA: 0.05<br>Similarity: 0.54	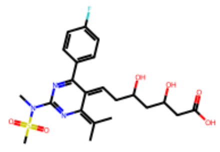<br>Exp HIA: 0.50<br>Pred HIA: 0.501577
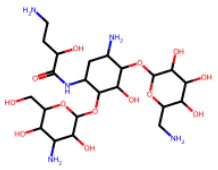<br>Exp HIA: 0.00<br>Similarity: 0.62	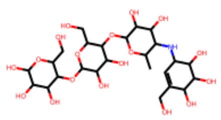<br>Exp HIA: 0.01<br>Pred HIA: 0.012653		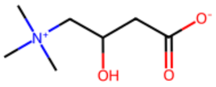<br>Exp HIA: 0.10<br>Similarity: 0.24	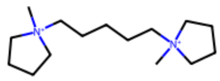<br>Exp HIA: 0.05<br>Pred HIA: 0.058325
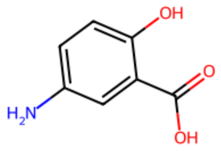<br>Exp HIA: 0.80<br>Similarity: 0.35	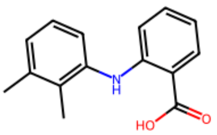<br>Exp HIA: 0.90<br>Pred HIA: 0.893194		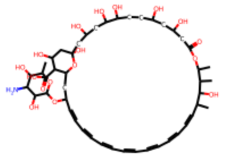<br>Exp HIA: 0.03<br>Similarity: 0.61	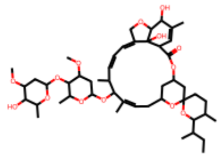<br>Exp HIA: 0.60<br>Pred HIA: 0.589562

Bottom3 predictions (highest AE values)

**Training set**	**Scaffold-based set**		**Training set**	**Least-similar set**
Mol in training	Mol in test		Mol in training	Mol in test
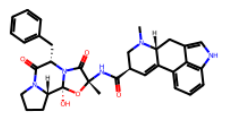<br>Exp HIA: 1.00<br>Similarity: 0.69	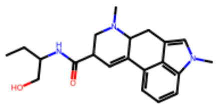<br>Exp HIA: 0.13<br>Pred HIA: 0.899020		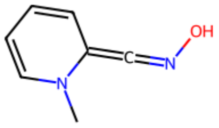<br>Exp HIA: 0.30<br>Similarity: 0.24	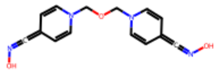<br>Exp HIA: 0.05<br>Pred HIA: 0.831893
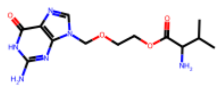<br>Exp HIA: 0.36<br>Similarity: 0.47	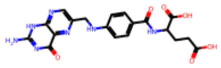<br>Exp HIA: 0.90<br>Pred HIA: 0.314903		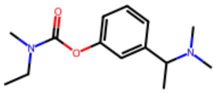<br>Exp HIA: 0.98<br>Similarity: 0.31	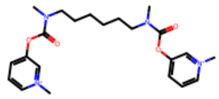<br>Exp HIA: 0.08<br>Pred HIA: 0.753090
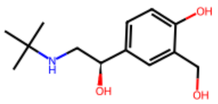<br>Exp HIA: 0.83<br>Similarity: 0.58	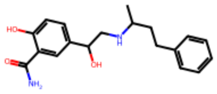<br>Exp HIA: 0.14<br>Pred HIA: 0.665275		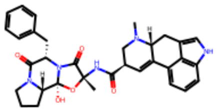<br>Exp HIA: 1.00<br>Similarity: 0.69	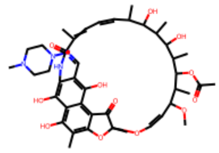<br>Exp HIA: 1.00<br>Pred HIA: 0.350335

Table S4. Property: logER ǀ ML algorithm: XTR

Top3 predictions (lowest AE values)

**Training set**	**Scaffold-based set**		**Training set**	**Least-similar set**
Mol in training	Mol in test		Mol in training	Mol in test
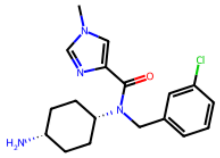<br>Exp logER: 0.176091<br>Similarity: 0.67	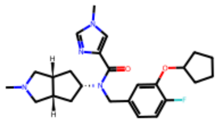<br>Exp logER: 0.447158<br>Pred logER: 0.446796		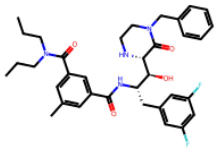<br>Exp logER: 1.230449<br>Similarity: 0.59	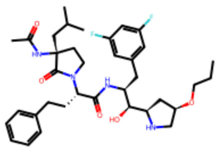<br>Exp logER: 1.079181<br>Pred logER: 1.079585
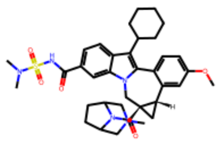<br>Exp logER: 0.342423<br>Similarity: 0.67	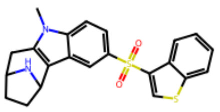<br>Exp logER: 0.447158<br>Pred logER: 0.448931		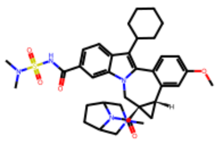<br>Exp logER: 0.342423<br>Similarity: 0.69	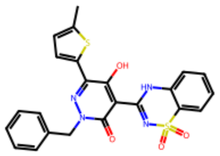<br>Exp logER: 0.778151<br>Pred logER: 0.765235
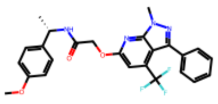<br>Exp logER: 0.000000<br>Similarity: 0.65	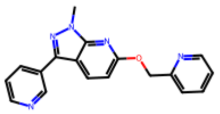<br>Exp logER: 0.053078<br>Pred logER: 0.061627		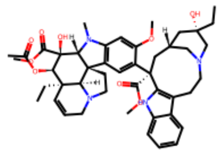<br>Exp logER: 0.255273<br>Similarity: 0.51	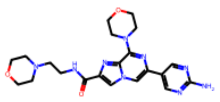<br>Exp logER: 0.732394<br>Pred logER: 0.710010

Bottom3 predictions (highest AE values)

**Training set**	**Scaffold-based set**		**Training set**	**Least-similar set**
Mol in training	Mol in test		Mol in training	Mol in test
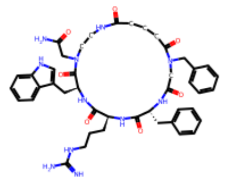<br>Exp logER: 0.477121<br>Similarity: 0.66	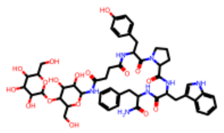<br>Exp logER: 2.845098<br>Pred logER: 0.339181		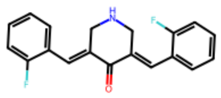<br>Exp logER: -0.221849<br>Similarity: 0.66	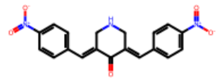<br>Exp logER: -2.000000<br>Pred logER: -0.025597
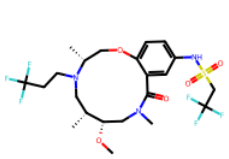<br>Exp logER: 0.181844<br>Similarity: 0.69	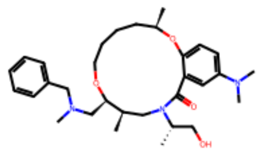<br>Exp logER: -0.552842<br>Pred logER: 0.874917		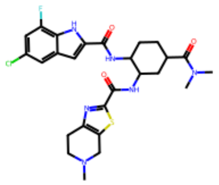<br>Exp logER: 1.477121<br>Similarity: 0.69	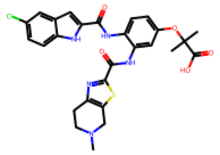<br>Exp logER: -0.619789<br>Pred logER: 1.081753
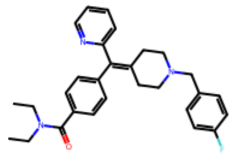<br>Exp logER: 0.342423<br>Similarity: 0.70	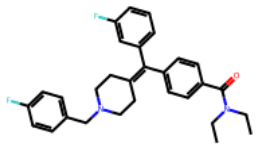<br>Exp logER: -1.000000<br>Pred logER: 0.344061		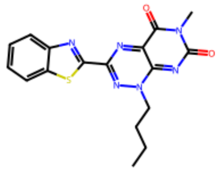<br>Exp logER: -1.522879<br>Similarity: 0.62	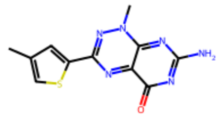<br>Exp logER: 1.691081<br>Pred logER: 0.137134

Table S5. Property: logBB | ML algorithm: XTR

Top3 predictions (lowest AE values)

Training set	Scaffold-based set		Training set	Least-similar set
Mol in training	Mol in test		Mol in training	Mol in test
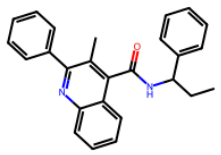 Exp logBB: 0.30<br>Similarity: 0.41	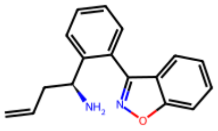 Exp logBB: 0.00<br>Pred logBB: -0.005228		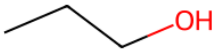 Exp logS: -0.15<br>Similarity: 0.67	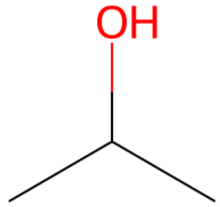 Exp logBB: -0.15<br>Pred logBB: -0.156872
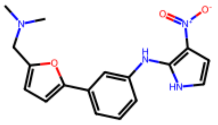 Exp logBB: -0.27<br>Similarity: 0.62	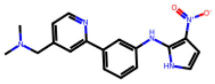 Exp logBB: -0.28<br>Pred logBB: -0.288964		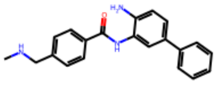 Exp logBB: -0.05<br>Similarity: 0.70	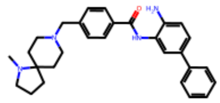 Exp logBB: -0.03<br>Pred logBB: -0.043777
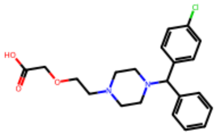 Exp logBB: -2.15<br>Similarity: 0.33	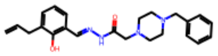 Exp logBB: -0.17<br>Pred logBB: -0.228645		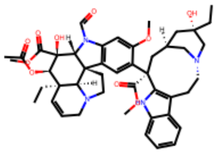 Exp logBB: -1.03<br>Similarity: 0.30	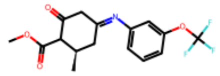 Exp logBB: -0.17<br>Pred logBB: -0.149555

Bottom3 predictions (highest AE values)

**Training set**	**Scaffold-based set**		**Training set**	**Least-similar set**
Mol in training	Mol in test		Mol in training	Mol in test
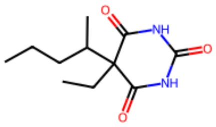<br>Exp logBB: 0.12<br>Similarity: 0.3	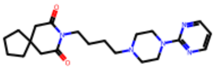<br>Exp logBB: 0.49<br>Pred logBB: -0.557531		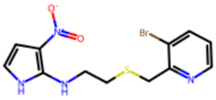<br>Exp logBB: -0.67<br>Similarity: 0.44	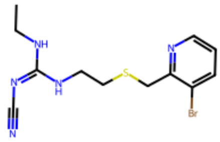<br>Exp logBB: -2.15<br>Pred logBB: -0.631670
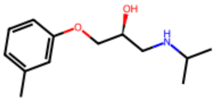<br>Exp logBB: 0.28<br>Similarity: 0.57	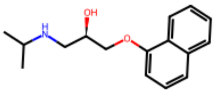<br>Exp logBB: 1.11<br>Pred logBB: 0.158374		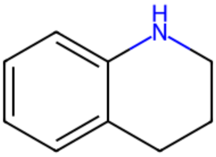<br>Exp logBB: 0.67<br>Similarity: 0.39	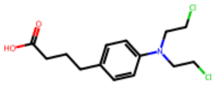<br>Exp logBB: -1.70<br>Pred logBB: -0.452960
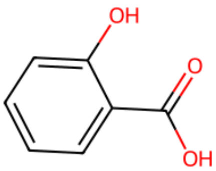<br>Exp logBB: -1.10<br>Similarity: 0.38	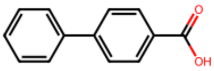<br>Exp logBB: -1.26<br>Pred logBB: -0.325086		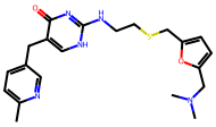<br>Exp logBB: -1.06<br>Similarity: 0.58	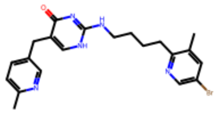<br>Exp logBB: -1.88<br>Pred logBB: -0.822481

Table S6. Property: PPB | ML algorithm: XTR

Top3 predictions (lowest AE values)

**Training set**	**Scaffold-based set**		**Training set**	**Least-similar set**
Mol in training	Mol in test		Mol in training	Mol in test
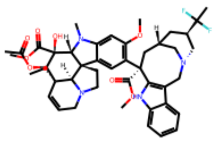 Exp PPB: 67.2<br>Similarity: 0.64	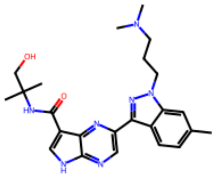 Exp PPB: 88.0<br>Pred PPB: 88.084603		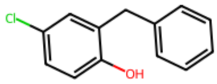 Exp PPB: 99.5<br>Similarity: 0.56	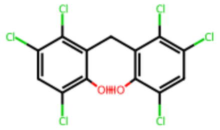 Exp PPB: 92.0<br>Pred PPB: 92.019892
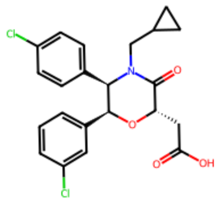 Exp PPB: 95.8<br>Similarity: 0.47	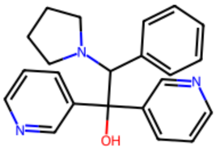 Exp PPB: 75.0<br>Pred PPB: 75.114210		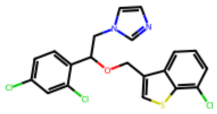 Exp PPB: 99.0<br>Similarity: 0.65	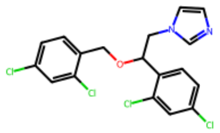 Exp PPB: 95.0<br>Pred PPB: 95.104153
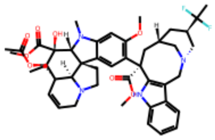 Exp PPB: 67.2<br>Similarity: 0.47	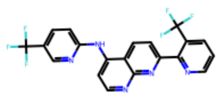 Exp PPB: 93.5<br>Pred PPB: 93.655072		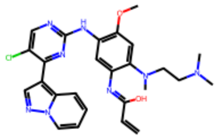 Exp PPB: 96.8<br>Similarity: 0.65	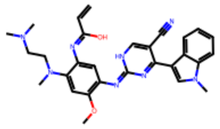 Exp PPB: 91.1<br>Pred PPB: 90.835724

Bottom3 predictions (highest AE values)

**Training set**	**Scaffold-based set**		**Training set**	**Least-similar set**
Mol in training	Mol in test		Mol in training	Mol in test
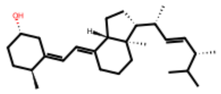<br>Exp PPB: 99.0<br>Similarity: 0.39	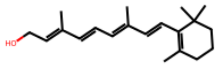<br>Exp PPB: 5.0<br>Pred PPB: 90.205418		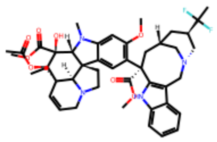<br>Exp PPB: 67.2<br>Similarity: 0.46	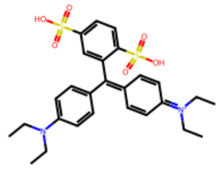<br>Exp PPB: 1.000<br>Pred PPB: 85.730251
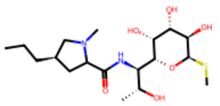<br>Exp PPB: 72.0<br>Similarity: 0.36	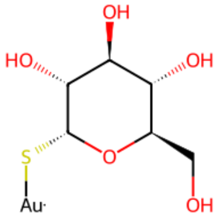<br>Exp PPB: 97.0<br>Pred PPB: 29.282845		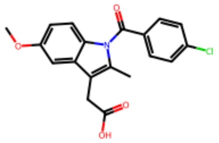<br>Exp PPB: 93.0<br>Similarity: 0.60	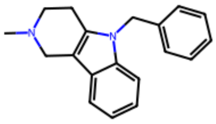<br>Exp PPB: 4.000<br>Pred PPB: 78.736977
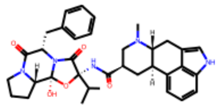<br>Exp PPB: 68.0<br>Similarity: 0.70	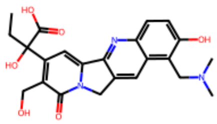<br>Exp PPB: 6.6<br>Pred PPB: 74.044217		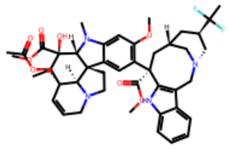<br>Exp PPB: 67.2<br>Similarity: 0.67	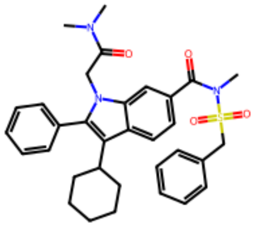<br>Exp PPB: 0.100<br>Pred PPB: 71.192453

Table S7. Property: logVDss | ML algorithm: XTR

Top3 predictions (lowest AE values)

**Training set**	**Scaffold-based set**		**Training set**	**Least-similar set**
Mol in training	Mol in test		Mol in training	Mol in test
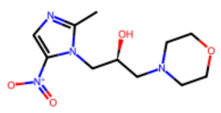<br>Exp logVDss: -0.124939<br>Similarity: 0.57	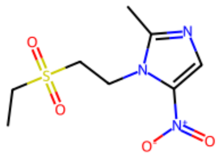<br>Exp logVDss: -0.229148<br>Pred logVDss: -0.226993		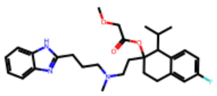<br>Exp logVDss: 0.491362<br>Similarity: 0.46	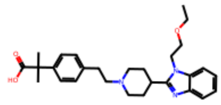<br>Exp logVDss: -0.214670<br>Pred logVDss: -0.209665
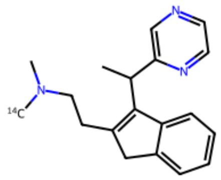<br>Exp logVDss: 0.269513<br>Similarity: 0.40	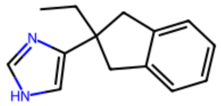<br>Exp logVDss: 0.431364<br>Pred logVDss: 0.434591		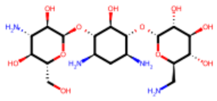<br>Exp logVDss: -0.585027<br>Similarity: 0.69	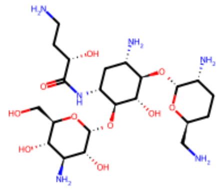<br>Exp logVDss: -0.677781<br>Pred logVDss: -0.684889
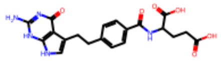<br>Exp logVDss: -0.823909<br>Similarity: 0.24	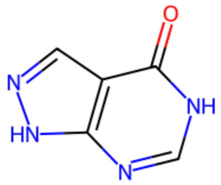<br>Exp logVDss: -0.236572<br>Pred logVDss: -0.240379		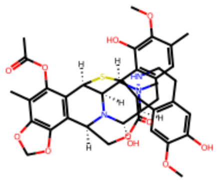<br>Exp logVDss: 1.397940<br>Similarity: 0.69	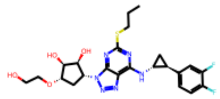<br>Exp logVDss: 0.100000<br>Pred logVDss: 0.109647

Bottom3 predictions (highest AE values)

**Training set**	**Scaffold-based set**		**Training set**	**Least-similar set**
Mol in training	Mol in test		Mol in training	Mol in test
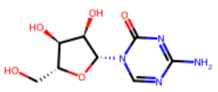<br>Exp logVDss: -0.327902<br>Similarity: 0.47	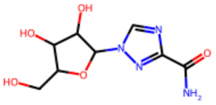<br>Exp logVDss: 1.146128<br>Pred logVDss: -0.150739		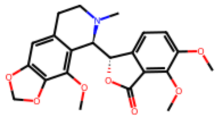<br>Exp logVDss: 0.361728<br>Similarity: 0.63	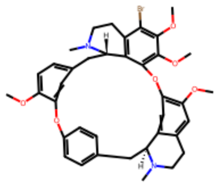<br>Exp logVDss: 1.707570<br>Pred logVDss: -0.062264
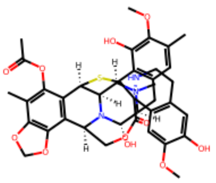<br>Exp logVDss: 1.397940<br>Similarity: 0.69	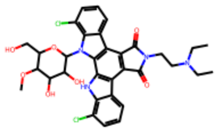<br>Exp logVDss: 0.949390<br>Pred logVDss: -0.213056		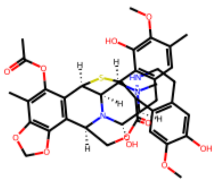<br>Exp logVDss: 1.397940<br>Similarity: 0.69	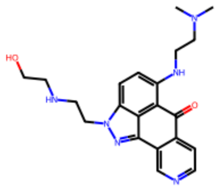<br>Exp logVDss: 1.755875<br>Pred logVDss: 0.256791
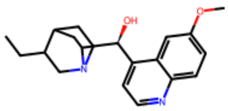<br>Exp logVDss: 0.450000<br>Similarity: 0.56	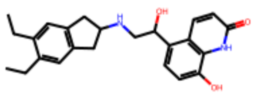<br>Exp logVDss: 1.287802<br>Pred logVDss: 0.272228		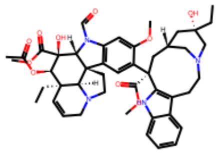<br>Exp logVDss: 0.380211<br>Similarity: 0.50	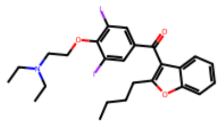<br>Exp logVDss: 1.778151<br>Pred logVDss: 0.288778

Table S8. Property: logCL | ML algorithm: XTR

Top3 predictions (lowest AE values)

**Training set**	**Scaffold-based set**		**Training set**	**Least-similar set**
Mol in training	Mol in test		Mol in training	Mol in test
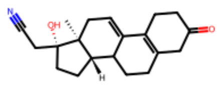 Exp logCL: 0.110590<br>Similarity: 0.59	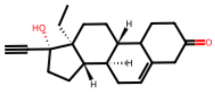 Exp logCL: 0.255273<br>Pred logCL: 0.246267		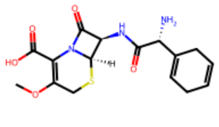 Exp logCL: 0.679428<br>Similarity: 0.70	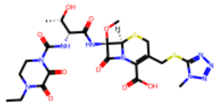 Exp logCL: 0.041393<br>Pred logCL: 0.032946
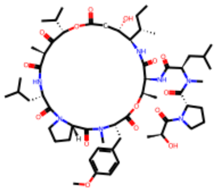 Exp logCL: 1.087071<br>Similarity: 0.49	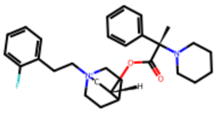 Exp logCL: -1.055517<br>Pred logCL: -1.075278		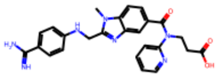 Exp logCL: 0.285557<br>Similarity: 0.57	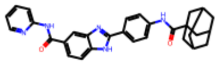 Exp logCL: -0.835647<br>Pred logCL: -0.859677
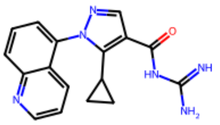 Exp logCL: 1.322219<br>Similarity: 0.52	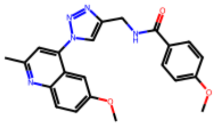 Exp logCL: -1.189969<br>Pred logCL: -1.163821		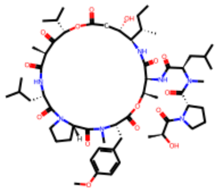 Exp logCL: 1.087071<br>Similarity: 0.63	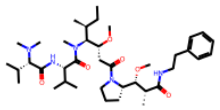 Exp logCL: 0.204120<br>Pred logCL: 0.230707

Bottom3 predictions (highest AE values)

**Training set**	**Scaffold-based set**		**Training set**	**Least-similar set**
Mol in training	Mol in test		Mol in training	Mol in test
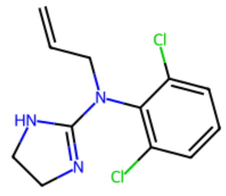<br>Exp logCL: 1.079181<br>Similarity: 0.46	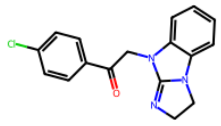<br>Exp logCL: -2.397940<br>Pred logCL: -0.164831		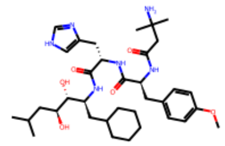<br>Exp logCL: -0.346787<br>Similarity: 0.62	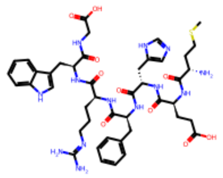<br>Exp logCL: 2.940018<br>Pred logCL: 0.124238
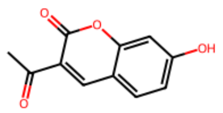<br>Exp logCL: -1.550059<br>Similarity: 0.50	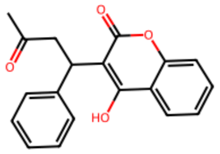<br>Exp logCL: -2.279841<br>Pred logCL: -0.195421		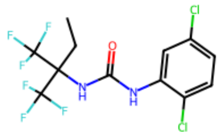<br>Exp logCL: -2.130182<br>Similarity: 0.07	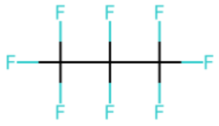<br>Exp logCL: 2.771374<br>Pred logCL: 0.074063
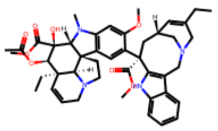<br>Exp logCL: 1.301030<br>Similarity: 0.53	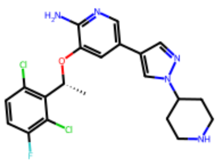<br>Exp logCL: 0.989005<br>Pred logCL: -1.053842		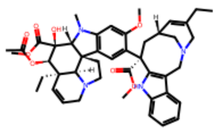<br>Exp logCL: 1.301030<br>Similarity: 0.48	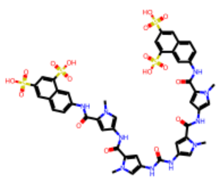<br>Exp logCL: -2.327902<br>Pred logCL: 0.040475

Table S9. Property: logHL ∣ ML algorithm: XTR

Top3 predictions (lowest AE values)

**Training set**	**Scaffold-based set**		**Training set**	**Least-similar set**
Mol in training	Mol in test		Mol in training	Mol in test
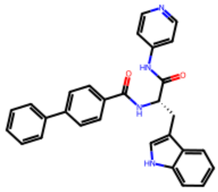 Exp logHL: -0.499350 Similarity: 0.67	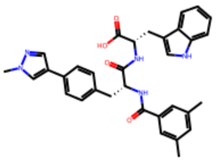 Exp logHL: 0.076094 Pred logHL: 0.077213		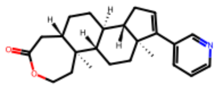 Exp logHL: -1.778060 Similarity: 0.60	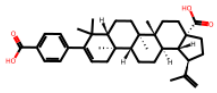 Exp logHL: 0.301030 Pred logHL: 0.301317
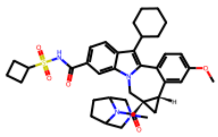 Exp logHL: 0.070038 Similarity: 0.69	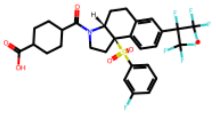 Exp logHL: 0.263162 Pred logHL: 0.264534		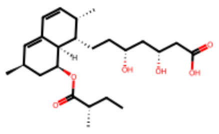 Exp logHL: 0.146128 Similarity: 0.52	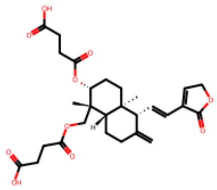 Exp logHL: 0.278754 Pred logHL: 0.280296
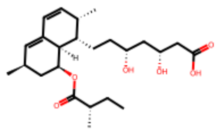 Exp logHL: 0.146128 Similarity: 0.52	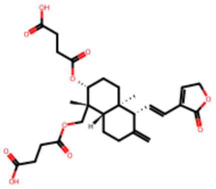 Exp logHL: 0.278754 Pred logHL: 0.280296		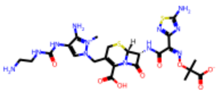 Exp logHL: 0.374582 Similarity: 0.67	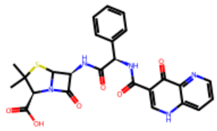 Exp logHL: 0.079181 Pred logHL: 0.081235

Bottom3 predictions (highest AE values)

**Training set**	**Scaffold-based set**		**Training set**	**Least-similar set**
Mol in training	Mol in test		Mol in training	Mol in test
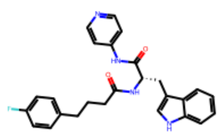<br>Exp logHL: -1.000000<br>Similarity: 0.67	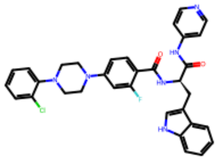<br>Exp logHL: -1.079200<br>Pred logHL: -0.362440		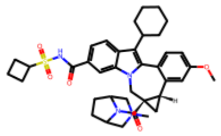<br>Exp logHL: 0.070038<br>Similarity: 0.60	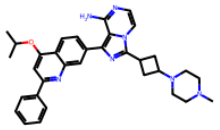<br>Exp logHL: 0.518514<br>Pred logHL: -0.255085
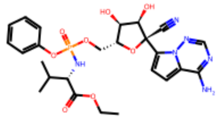<br>Exp logHL: 1.421604<br>Similarity: 0.70	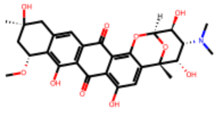<br>Exp logHL: 1.121231<br>Pred logHL: 0.424448		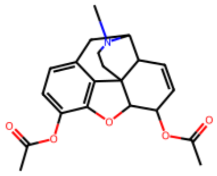<br>Exp logHL: 0.397940<br>Similarity: 0.70	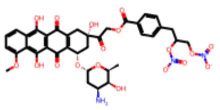<br>Exp logHL: 1.204120<br>Pred logHL: 0.473431
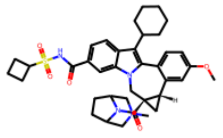<br>Exp logHL: 0.070038<br>Similarity: 0.66	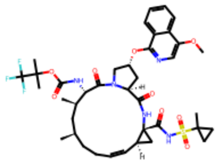<br>Exp logHL: -0.560670<br>Pred logHL: 0.112890		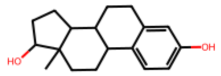<br>Exp logHL: 0.230449<br>Similarity: 0.67	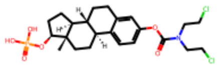<br>Exp logHL: 0.380211<br>Pred logHL: -0.317195
